# Association of Rare Immune-Related Adverse Events to Survival in Advanced Cancer Patients Treated with Immune Checkpoint Inhibitors: A Real-World Single-Center Cohort Study

**DOI:** 10.3390/cancers14092276

**Published:** 2022-05-03

**Authors:** Saara Kuusisalo, Jussi P. Koivunen, Sanna Iivanainen

**Affiliations:** Department of Oncology and Radiotherapy, Oulu University Hospital and MRC Oulu, 90220 Oulu, Finland; saara.kuusisalo@student.oulu.fi (S.K.); jussi.koivunen@ppshp.fi (J.P.K.)

**Keywords:** immune checkpoint inhibitors, immune-related adverse event, prognostic, survival, autoimmunity

## Abstract

**Simple Summary:**

Immune checkpoint inhibitors (ICIs), while having changed the treatment of multiple cancers, come with novel immune-related adverse events (irAEs) resembling autoimmune diseases. The registration data suggest that at least some irAEs have a prognostic nature regarding the efficiency of ICIs. However, real-world data on the matter are scarce and partly controversial. Moreover, the role of ethnicity and sub-population genetics in affecting the immune system in ICI outcomes regarding efficiency and toxicity warrants more research.

**Abstract:**

Immune checkpoint inhibitors (ICIs) are associated with immune-related (ir) adverse events (AEs) resembling autoimmune diseases. In this retrospective cohort study of patients (pts) treated with ICIs at Oulu University Hospital from 2014–2020, we analysed the spectrum of severe irAEs and their prognostic nature, focusing on rare irAEs. Pts (*n* = 173) with lung cancer (*n* = 76, 43.9%), melanoma (*n* = 56, 32.4%), renal and bladder cancers (*n* = 34, 19.7%), head and neck cancers (*n* = 4, 2.3%), SCC (*n* = 2, 1.2%), and CRC (*n* = 1, 0.6%) receiving single anti-PD-(L)1 (*n* = 160) or combination (ICI-ICI *n* = 9, ICI-chemotherapy *n* = 4) therapy were included. The survival analysis focused on single anti-PD-(L)1-treated patients with melanoma, lung cancer, and renal and bladder cancers (*n* = 142). Grade ≥ 3 irAEs of multiple aetiology occurred in 29 patients treated with single-PD-L1 therapy (20.4%), which was associated with improved progression-free survival (PFS) (HR 0.50, CI 0.31–0.78) but not overall survival (OS) (HR 0.88, CI 0.52–1.50). Rare grade ≥ 3 events occurred in 10 (7.0%) pts with no association with PFS (HR 0.90, CI 0.42–1.94). Hence, the presence of rare grade ≥ 3 irAEs was associated with a tendency for inferior OS (HR 1.44, CI 0.66–3.11). Pts with rare grade ≥ 3 irAEs had inferior OS, possibly reflecting the delay in diagnostic workflow and the treatment of irAEs. One explanation for the high incidence of irAEs could be the Finnish population-based genetic variation affecting the immune system.

## 1. Introduction

Immune checkpoint inhibitor therapies (ICIs) targeting immune checkpoints such as cytotoxic T-lymphocyte-associated antigen 4 (CTLA-4), programmed cell death protein (PD-1), and its ligand (PD-L1) have produced long-term tumor responses in multiple advanced cancers [1,2,3,4,5,6,7,8,9,10,11,12]. Checkpoint blockade has been proven to induce immune-related adverse events (IrAEs) as novel treatment-related adverse events (TRAEs) compared to traditional cancer medications [13]. The rationale behind the toxicities of ICIs is to block the physiological role of the checkpoint pathways in regulating adaptive immunity and inhibiting autoimmunity [14]. Therefore, IrAEs often manifest similarly to autoimmune diseases [15]. ICI treatments are associated with a wide variety of TRAEs, the most common of which are rashes, endocrinological changes, and inflammation of the intestines, lungs, and liver. Approximately 15% of patients receiving ICI monotherapies have been reported to have had severe grade 3–4 side effects; about 30% face lower-grade adverse events (AEs) [16]. Rare side effects include, among others, changes in blood counts, myocarditis, and neurological syndromes [17]. However, the data are limited regarding clinical outcomes or toxicity of ICIs by ethnicity, which might carry an impact considering the interplay between host immunity and tumors in the sense of adaptive immunity [18].

The AEs of medical therapies are classified according to frequency and severity based on the NCI Common Terminology Criteria for Adverse Events (CTCAE) criteria. The international guidelines for managing IrAEs are based on the CTCAE grading and include the guidance on using supportive medications, mainly immunosuppressives, and advice on continuing the cancer drug. Grade 3 to 4 IrAEs are perceived as severe adverse reactions, typically resulting in permanent discontinuation of the immunological therapy.

Patients with underlining autoimmune diseases are mostly excluded from the registration trials of ICIs. The scarce data suggest that some patients develop flare-ups of autoimmune disease (AD) and IrAEs with higher frequency while on ICI therapy, which can be associated with excess morbidity [19]. The incidence of autoimmune disorders in Finnish ethnicity is the highest in the world for type I diabetes and among the highest for celiac disease. There is also population-based evidence of excess risk of concomitant ADs [20,21,22]. No reported trials are investigating the spectrum of IrAEs among cancer patients treated with ICIs in Finland. Interestingly, previous data have suggested that dermatological and endocrinological irAEs are associated with improved survival in patients treated with ICIs [23,24,25,26,27].

This study aims to evaluate the spectrum of IrAEs in a real-world population with a high incidence of autoimmunity and the survival relative to the prevalence and nature of IrAEs. We hypothesized that the frequency and spectrum of IrAEs in our study population would differ from the incidence reported in ICI registration trials. Even global registration trials might be unable to capture the differences in the side effect profile of a drug related to ethnical differences. Therefore, population studies could bring additional information to the topic.

## 2. Materials and Methods

All patients who received at least one dose of intravenous immune checkpoint inhibitor therapy as a single therapy or combination therapy at Oulu University Hospital from 2014–2020 were retrospectively identified from the pharmacy records. The patients who had received the treatment as an adjuvant therapy after curative surgery were formed into a separate cohort. Demographic data, such as the patient’s age, date of diagnosis, date of advanced/metastatic disease, TNM staging, histology, the molecular status of the tumor, the treatment regimens, the treatment-related adverse events, tumor responses, date of progression, and date of death/last follow-up, were collected manually from the electronic health record (EHR). Progression-free survival (PFS) and overall survival (OS) were calculated from the first date of ICI treatment to the documented tumor progression, death, or end of follow-up (PFS) or to death or end of follow-up (OS). Disease-free survival (DFS) was calculated from the first date of ICI treatment to the documented disease recurrence, death, or end of follow-up. Tumor progression, disease recurrence, and/or death were counted as an event or events.

Prospectively recorded IrAEs were collected from the EHR and graded according to CTCAE criteria. The nature, date of IrAE, length of IrAE, and possible treatment of IrAE were registered. The focus was on severe grade 3–5 IrAEs as the milder IrAEs were not comprehensively collected. The IrAE events were collected by two investigators (SK and SI). If no consensus on the event or its grading was reached, the third investigator (JPK) evaluated the event, which was recorded according to the majority consensus. The frequency of TRAES was listed according to the following classifications: common toxicities arise at the rate of >1% (>1 in 100), uncommon toxicities of 1% to 0.1% (1 in 100 to 1 in 1000), rare toxicities at a rate of 0.1% to 0.01% (1 in 1000 to 1 in 10,000), and very rare toxicities at a rate of less than 0.01% (<1:10,000) (WHO, World Health Organization).

The data collection was carried out according to national legislation and under a permit from the medical director of Oulu University Hospital (study no. 299/2016). Pseudonymization was carried out before data analysis. Informed consent was not sought due to the register nature of the study.

IBM SPSS Statistics 27.00.00 for Windows was applied for statistical analysis. Survival was analyzed with the Kaplan–Meier and Cox regression methods, with 95% confidence intervals.

## 3. Results

### 3.1. Patients Receiving ICI Therapy

A total of 173 patients treated with ICI therapy for various advanced cancers from 2014–2020 were included in the analysis. The median age of the patients was 66; most of the patients (*n* = 122, 70.5%) were male, and only three had a history of distinct autoimmune disease. The cohort included patients with lung cancer (non-small cell lung cancer, NSCLC) (*n* = 76, 43.9%), melanoma (*n* = 56, 32.4%), renal and bladder cancers (genitourinary, GU) (*n* = 34, 19.7%), head and neck cancers (*n* = 4, 2.3%), squamous cell carcinoma (SCC) (*n* = 2, 1.2%), and colorectal cancer (CRC) (*n* = 1, 0.6%). In our cohort, 13 received combination therapy, nine anti-PD-(L)1-CTLA-4 regimens, four anti-PD-(L)1 plus chemotherapy, and the rest (*n* = 160) single-anti-PD-(L)1 treatment. At the start of the ICI therapy, 91 (52.6%) of the patients had performance status ECOG 1, 75 (43.4%) ECOG 0, and 7 (4.0%) ECOG 2, while 143 (82.7%) patients had stage IV disease; the rest of the population had stage III disease (*n* = 30, 17.3%). Sixty-seven (38.7%) patients received IO-therapy as a first-line treatment, 73 (42.2%) as a second-line, 14 (8.1%) as a third-line, and 8 (4.6%) in a fourth or later line. Eleven patients (6.3%) received anti-PD-1 therapy as an adjuvant treatment (Table 1). The median (m) follow-up time was 12 months (mo.) (ranging from 0–78 mo.), and the median length of therapy was 3.2 months (0–77 mo.). The mPFS was 4.53 months for the whole cohort, and the mOS was 13.96. DFS was not reached (Table 1). The prognosis in advanced melanoma differs greatly from the prognosis of NSCLC. According to the SEER database, the five-year survival rate for stage IV melanoma is 16%, while in NSCLC the rate is 7%. Moreover, around 40% of melanoma patients respond to ICI, while in NSCLC the rate is around 20%. We planned to analyze PFS and OS separately in these two diseases—the two largest cancer types in our cohort—to control these differences in baseline factors. However, there was no statistically significant difference in mPFS based on the stratification by melanoma (5.09 mo.) or non-small cell lung cancer (4.90 mo.) (*p* = 0.055, not shown). Thus, we formed two cohorts—melanoma and other disease types (Table 1). The mPFS for patients with NSCLC and other tumor types (*n* = 117) was 4.40 mo.; for melanoma patients, it was (*n* = 45) 5.09 mo. (HR 0.62, CI 0.41–0.95; *p* = 0.027), while no statistically significant difference in OS was seen.

### 3.2. Immune-Related (ir) Gr3–5 Adverse Events (AEs), PFS and OS

Grade 3 or higher irAEs occurred in 42 of 173 patients (24.2%), and the total number of irAEs was 60. Eight patients who experienced grade ≥ 3 irAE received combination therapy, three ICI-chemo, and five ICI-ICI. Of the 42 patients, 21 had lung cancer (irAE incidence 27.6%), 12 melanoma (irAE incidence 21.4%), six GU cancer (irAE incidence 17.6%), two SCC, and one CRC. The incidence of irAEs varied among the patient cohort; 29 (16.8%) experienced one, eight (4.6%) experienced two, and five (2.9%) experienced three separate irAEs. The median time to the first occurrence of irAEs from the start of the therapy was 2.0 mo., with a corresponding mean time of 3.2. mo. (range 0–16 mo.). Most (*n* = 26, 61.9%) of the patients experienced irAEs during the treatment. Grade 3–5 irAEs were of different tissue aetiology, e.g., skin, gastrointestinal, and lung toxicity, as described in detail in Table 2.

The cohort of PD-1 adjuvant therapy consisted of 11 patients, two of whom developed grade 3 or higher irAEs (18.2%), suggesting that AE frequency is similar to the non-curative setting. However, the small sample size does not enable a more detailed analysis of this cohort.

**Figure 1:**
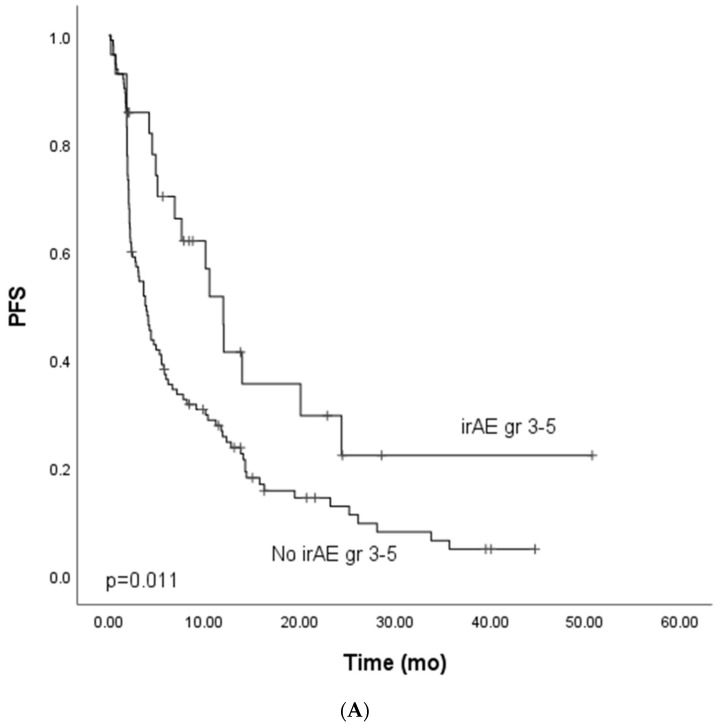

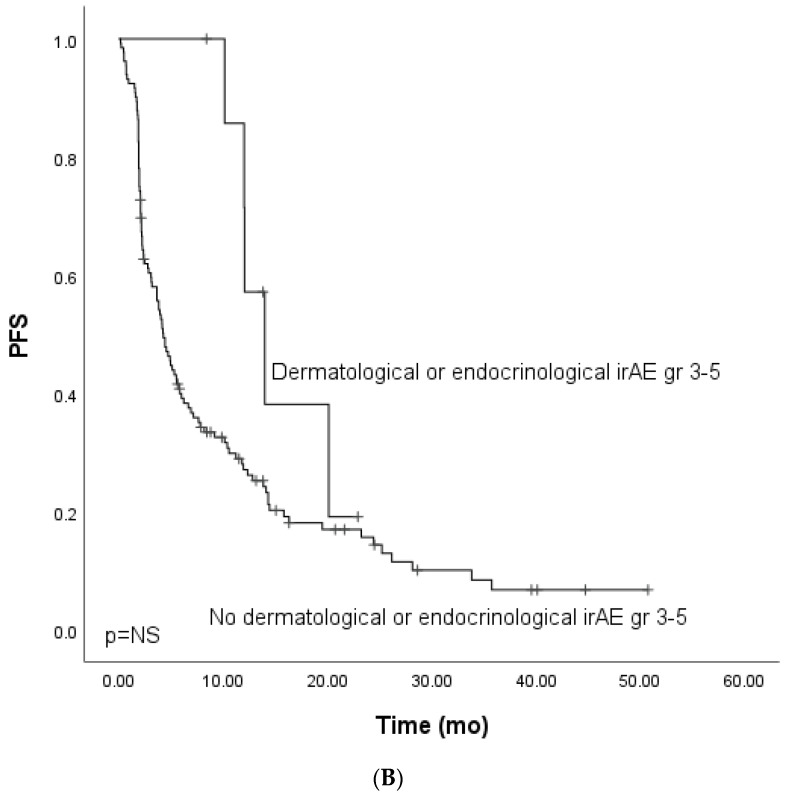

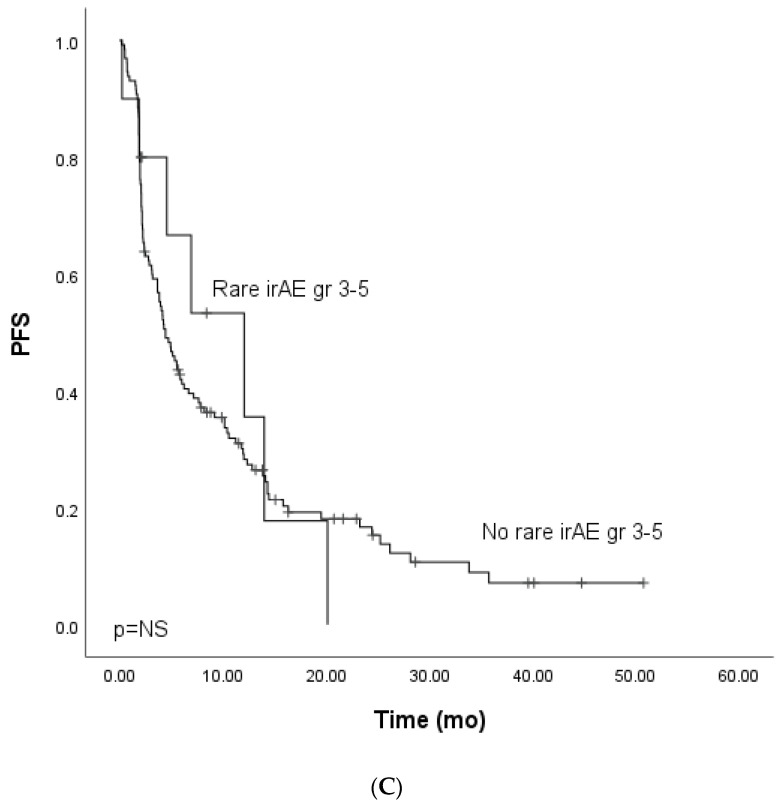
Kaplan–Meier analysis for progress-free survival for patients with (**A**) grade ≥ 3 immune-related adverse events; (**B**) grade ≥ 3 dermatological or endocrinological immune-related adverse events; (**C**) rare grade ≥ 3 immune-related adverse events. Crosses indicate censored events.

**Figure 2:**
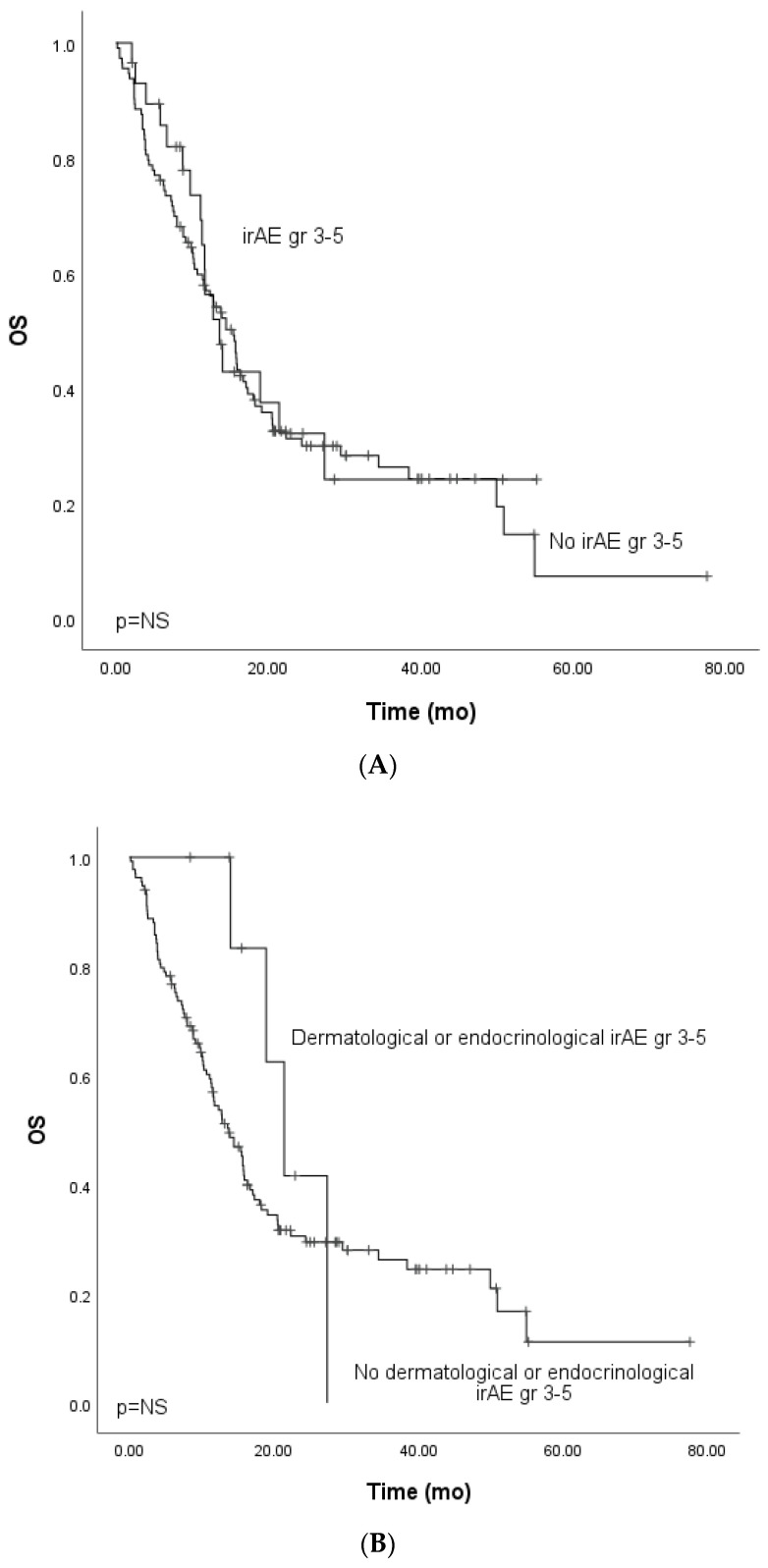

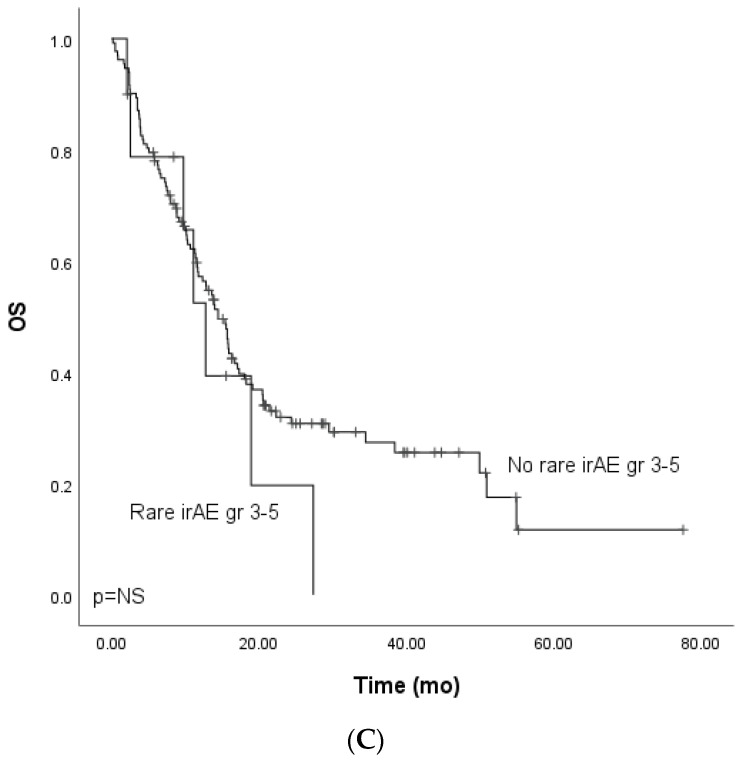
Kaplan–Meier analysis for the overall survival for patients with (**A**) grade ≥ 3 immune-related adverse events; (**B**) grade ≥ 3 dermatological or endocrinological immune-related adverse events; (**C**) rare grade ≥ 3 immune-related adverse events. Crosses indicate censored events.

We focused on the non-curative patients with melanoma, lung cancer, and GU cancers with single-PD-(L1) therapy (*n* = 142) in the survival analysis to control confounding factors. In univariate analysis, grade ≥ 3 irAEs were associated with improved PFS. In multivariate analysis, including tumor type (melanoma vs. other), ECOG (0 vs. other), presence of grade ≥ 3 irAEs, and peripheral blood C-reactive protein (CRP) level (under or ≥10 mg/L), the association between improved PFS and grade ≥ 3 irAEs was retained (Table 3, Figure 1A). In the univariate analysis for OS, grade ≥ 3 irAEs (0.88, CI 0.52–1.50, Figure 2A) were not associated with improved survival.

### 3.3. Immune-Related Gr3–5 Dermatological and Endocrinological Adverse Events, PFS, and OS

As previous works have linked endocrinological and dermatological irAEs to prognosis, we analyzed these irAE categories in the cohort. Grade ≥ 3 dermatological or endocrinological immune-related adverse events occurred in 15 (9.3%) patients. The total number of grade ≥ 3 dermatological or endocrinological irAEs was 19. Dermatological and endocrinological adverse events included dermatotoxicity (*n* = 6, 31.6%), hypothyroidism (*n* = 5, 26.3%), hypophysitis (*n* = 5, 26.3%), adrenal cortex insufficiency (*n* = 2, 10.5%), and bullous dermatitis (*n* = 1, 5.3%). The mPFS for patients with dermatological or endocrinological adverse events did not differ from that of patients with irAEs of another subtype (HR 0.44, Cl 95% 0.18–1.1) (Figure 1B).

The OS analysis for patients with dermatological or endocrinological irAEs showed a tendency for improved survival (HR 0.56, CI 95% 0.21–1.53). However, the OS did not reach clinical significance, probably due to the cohort’s small size (Figure 2B).

### 3.4. Immune-Related Gr3–5 Rare Adverse Events, PFS, and OS

We further analyzed the rare (<1% incidence in the summary of product characteristics of nivolumab or pembrolizumab) grade ≥ 3. Rare grade ≥ 3 events occurred in 16 (9.9%) patients (lung cancer, *n* = 8, melanoma, *n* = 4, GU cancers, *n* = 3, SCC, *n* = 1). The total number of rare irAEs was 19. Five patients who experienced rare grade ≥ 3 IrAEs received combination therapy (two ICI-chemo and three ICI-ICI). Rare AEs included hypophysitis (*n* = 5, 3.1%), adrenal cortex insufficiency (*n* = 2, 1.2%), anaphylaxis (*n* = 2, 1.2%), diabetes mellitys type 1 (*n* = 2, 1.2%), cholangitis (*n* = 2, 1.2%), dermatotoxicity (pemfigoid, *n* = 1), bullous dermatitis (*n* = 1), nephritis (*n* = 1), immune thrombocytopenia (*n* = 1), polyneuropathy (*n* = 1), and rhabdomyolysis (*n* = 1). Due to the small number of patients in the cohort of combination therapy, survival analysis focused on patients treated with single-PD-(L)1 therapy, which included patients with melanoma, lung cancer, and GU cancers. There was no statistically significant difference in mPFS (HR 0.90, CI 95% 0.42–1.94) or mOS between the groups (HR 1.44, CI 95% 0.66–3.11) (Figure 1C and Figure 2C).

## 4. Discussion

Global registration trials might not capture a drug’s ethnicity-related side effect profiles. Our study results confirmed the hypothesis that the spectrum of severe ICI-induced TRAEs in our real-world cohort of multiple-type cancer patients differs from that in the registration trials. Similarly, ethnicity-associated immune-mediated side effect profiles have been detected, e.g., with H1N1 vaccines and their relation to childhood narcolepsy in specific ethnic population isolates such as in Finland [28,29].

According to our data, PD-1 or PD-L1 inhibition causes high-grade irAEs in 10–15% of patients, with similar incidences seen with different agents in a dose-independent fashion, while with ICI-ICI combination therapies the rate of grade ≥ 3 irAEs is as high as 50%. On the contrary, the rate of irAEs in single-ICI-chemotherapy treatment follows the toxicity profile of anti-PD-(L)1 monotherapies [16,17]. Furthermore, TRAEs regarded as rare or uncommon based on ICI registration studies were more common (9.9%) in our real-world evidence (RWE) study [30,31]. The rather high incidence of severe rare endocrinological irAEs was particularly notable.

RWE studies of ICI TREAs are scarce, but some investigators have identified that TRAEs are more common in RWE settings [32,33]. The overall incidence of irAEs and endocrinological irAEs is higher with immunotherapy combinations, including combinations of anti-PD-(L)1 and anti-CTLA-4 [34,35]. In our study, the incidence of severe irAEs among those treated with combination therapies was higher (61.5%) compared to that of the monotherapy-treated patients (21.5%). However, the frequency of grade ≥ 3 irAEs in the monotherapy group was still significantly higher than in the registration studies. The incidence of rare severe irAEs was especially high in our cohort, with 32.8% of the identified grade ≥ 3 irAEs classified as rare in the whole cohort, and 20.8% in the cohort of single-PD-(L)1-treated patients with melanoma, lung cancer, or GU cancer [30,31].

We speculate that the high incidence of irAEs is most likely associated with study-specific, patient-related factors. This study was performed at a single center with a quite specific population of Finnish patients. However, our patient cohort closely resembles the I/E criteria of the ICI registration studies, where underlying auto-immunity (excluding hypothyroidism, DM1, vitiligo, and celiac disease) was very scarce. According to the previous data, pre-existing autoimmune disease prones cancer patients to develope irAEs [19]. While the prognostic role of the human leukocyte antigen (HLA) class I-dependent immune activity is linked to autoimmune diseases, and HLA class I-dependent CD8+ T cells are required for immune checkpoint blockade anti-tumor activity, it is poorly known whether HLA class I is predictive of toxicity to ICIs [36,37,38]. Finland has the highest incidence of type I diabetes (T1D) in children (62.5 per 100,000) and is among the countries with the highest incidence of celiac disease in young adults (31.0 per 100,000) in the world [21,22]. Interestingly, a recent large Finnish nationwide cohort study revealed that autoimmune diseases (ADs) are more often present in individuals with T1D and come with an excess risk of concomitant ADs [23]. One explanation for the high incidence of irAEs in our cohort could be the population-based genetic variation affecting the immune system in Finnish individuals constituting a Northern European genetic isolate.

A recent article by Lozano et al. showed, by combining in vitro and clinical data, that two pre-treatment T cell characteristics, activated CD4 Tm cell abundance, and a more clonally diverse TCR repertoire in the peripheral blood were linked to ICI-induced irAEs in patients with metastatic melanoma. Interestingly, they also observed elevated levels of activated CD4 Tm cells in patients with SLE or IBD, implying that severe irAEs might represent a subclinical or latent autoimmune state that is clinically unmasked on ICI administration [39]. The authors concluded that a common immunological mechanism underlying irAE development and autoimmunity is possible.

As with some previous studies, we found an association between irAEs and improved treatment outcomes [23,24,25,26,27]. Grade ≥ 3 irAEs were associated with improved PFS in univariate and multivariate analysis. Surprisingly, the observed PFS advantage did not translate to improved overall survival, although similar findings have been observed [40]. With cancer immunotherapies, discordance between PFS and OS has been observed in some registrational studies (CheckMate 025, CheckMate 057, Keynote 048), with improved OS and non-altered PFS. The prognostic role of irAEs regarding the incongruence in the relation of PFS to OS might differ in the sense that they often require treatment, and suboptimal AE management could sacrifice the treatment benefit. While the possibility that the immune activation underlying most irAEs might be paired with the activity required for anti-tumor immune responses is speculated, the evidence suggests that some irAEs might have mechanisms unrelated to anti-tumor activity, including those involving the microbiome and viral or tissue-specific factors [41,42,43]. Furthermore, the growing data imply that the aetiology of acute irAEs might differ from chronic/irreversible ICI-related toxicities [44]. It is likely that irAEs arise from both tumor-related and tumor-unrelated features.

Given the considerable heterogeneity of ICI-induced irAEs, including variations in their timing and location, one possible factor related to the inconsistency between PFS and OS in our cohort could be the inadequate detection and treatment of TRAEs. A retrospective study with a large number of subjects (*n* = 13,030) showed that irAEs requiring inpatient visits are linked to poor outcomes and higher mortality [45]. We speculate that PFS not being a surrogate for OS in grade ≥ 3 irAE patients could likely relate to the lack of prompt recognition and treatment of irAEs, and this cannot be ruled out. We observed only two deaths related to irAEs in our cohort, which cannot explain the discordance between PFS and OS. However, the data cannot reveal whether suboptimal management of irAEs could lead to an increased risk of cancer-related deaths. The observed large spectrum of irAEs rising basically from all organ systems makes recognizing TRAEs difficult, especially in rural areas where the distances between the patients’ primary healthcare unit and the tertiary oncology centers are long, such as in Northern Finland. Altogether, we speculate that traditional baseline factors might not explain the observed results. Multiple confounding factors with retrospective prognostic assessment exist, the major factor being the time-related bias where patients with a favorable overall prognosis have more time to experience the treatment’s different side effects. However, in our cohort, the median time to the first occurrence of grade 3–5 irAEs from the start of the therapy was two months. Thus, most of the patients experienced a severe treatment-related adverse event in the early phase of the treatment, which seems typical with severe irAEs [46]. One cannot overlook the rather small sample size in creating statistical uncertainties. We aimed to control the source of bias with parallel statistical methods in survival analysis. Furthermore, our patient cohort was sufficiently homogenous. Thus, ~90% of patients received ICIs as a first- or second-line therapy, and 95.7% were in excellent or good performance status. In addition, the survival analysis focused on single-PD-(L)1-treated patients of the three biggest disease cohorts (melanoma lung cancer, and GU cancers). Some of the patients (*n* = 15) experienced late severe irAEs after discontinuing the therapy. The reason for treatment discontinuation was disease progression in only two patients. For the remaining patients, suspected/confirmed lower grade irAEs or the institutional maximal therapy length (6 mo.) led to the discontinuation of the therapy.

## 5. Conclusions

Our results show that the severe toxicities of ICIs in monotherapies and combinations are more common and come with a wider spectrum in clinical practice than in registration studies. Our results also show that at least some of the TRAEs have a prognostic nature (PFS) in multiple advanced cancer types. Promptly recognizing and treating severe irAE is warranted to unleash the full therapeutic potential of ICIs. Thus, new tools should be developed for recognizing irAEs. Moreover, the role of ethnicity and sub-population genetics affecting the immune system in ICI outcomes in efficiency and toxicity warrants more research.

## Figures and Tables

**Table 1:** Patient demographics.

Age (Median, Years)	*n* (%)
Age (median), years	66
Sex	
Male	1226 (70.5)
Female	51 (29.5)
Previous autoimmunity (excluding celiac, vitiligo, hypothyroidism, DM1)	3 (1.7)
Tumor type	
Lung, non-small cell	76 (43.9)
Melanoma	56 (32.4)
GU	34 (19.7)
Head and neck	4 (2.3)
SCC	2 (1.2)
Colorectal	1 (0.6)
Stage at diagnosis	
IV	143 (82.7)
III	30 (17.30)
ECOG performance status	
0	75 (43.4)
1	91 (52.6)
2	7 (4.0)
Monotherapy	160 (92.5)
Combination therapy	13 (7.5)
Line of treatment	
First	67 (38.7)
Second	73 (42.2)
Third	14 (8.1)
Later	8 (4.6)
Adjuvant	11 (6.4)
PFS time (median), months (non-curative)	4.53
OS time (median), months (non-curative)	13.96
DFS time (median), months (curative)	Not reached

Values are presented as *n* (%) unless indicated otherwise. GU, genitourinary (renal and bladder); SCC, squamous cell carcinoma.

**Table 2:** Grade 3–5 immune-related (ir) adverse events in the whole cohort.

Spectrum of irAES	*n* (%)
Treatment-related irAEs (patients)	
Yes	42 (24.2)
PD-(L)1 monotherapy	34 (81)
Combination	8 (190)
No	131 (76.9)
Frequency of irAEs	
1	29 (16.8)
2	8 (4.6)
3	5 (2.9)
Total number of irAEs	60
Grade (gr) of irAE	
3	42 (70.0)
4	15 (25.0)
5	2 (3.3)
Type of irAE	
Skin	6 (10.0)
Endocrinological	5 (8.3)
Hepatotoxicity	9 (15.0)
GI toxicity	8 (13.3)
Pneumonitis	11 (18.3)
Musculoskeletal	2 (3.3)
Rare	19 (31.7)
Median time to first gr ≥ 3 IrAE occurrence (months)	2.0
First gr ≥ 3 irAE occurrence on ICI therapy	
Yes	26 (61.9)
No	16 (38.1)

Values are presented as *n* (%) unless indicated otherwise. irAE, immune-related adverse event; ICI, immune checkpoint inhibitor.

**Table 3:** Univariate and multivariate analysis for progression-free survival according to the presence of grade ≥ 3 irAE or grade ≥ 3 endocrinological or dermatological irAE.

Features	Univariate		Multivariate	
	HR	CI (95%)	HR	CI (95%)
Grade ≥ 3 irAE				
Yes vs. No	0.50	0.31–0.87	0.52	0.29–0.95
Disease type				
Melanoma vs. Other	0.57	0.37–0.88	0.94	0.53–1.68
ECOG				
0 vs. 1–2	0.53	0.36–0.79	0.60	0.36–0.99
Peripheral blood CRP level				
under or ≥10 mg/L	0.46	0.30–0.72	0.58	0.35–0.97

## Data Availability

The datasets generated and/or analyzed during the current study are not publicly available. However, they are available from the corresponding author upon reasonable request.

## References

[1] Topalian S.L., Hodi F.S., Brahmer J.R., Gettinger S.N., Smith D.C., McDermott D.F., Powderly J.D., Carvajal R.D., Sosman J.A., Atkins M.B. (2012). Safety, activity, and immune correlates of anti-PD-1 antibody in cancer. N. Engl. J. Med..

[2] Schachter J., Ribas A., Long G.V., Arance A., Grob J.J., Mortier L., Daud A., Carlino M.S., McNeil C., Lotem M. (2017). Pembrolizumab versus ipilimumab for advanced melanoma: Final overall survival results of a multicentre, randomised, open-label phase 3 study (KEYNOTE-006). Lancet.

[3] Robert C., Schachter J., Long G.V., Arance A., Grob J.J., Mortier L., Daud A., Carlino M.S., McNeil C., Lotem M. (2015). Pembrolizumab versus Ipilimumab in Advanced Melanoma. N. Engl. J. Med..

[4] Weber J.S., Hodi F.S., Wolchok J.D., Topalian S.L., Schadendorf D., Larkin J., Sznol M., Long G.V., Li H., Waxman I.M. (2017). Safety Profile of Nivolumab Monotherapy: A Pooled Analysis of Patients With Advanced Melanoma. J. Clin. Oncol..

[5] Borghaei H., Paz-Ares L., Horn L., Spigel D.R., Steins M., Ready N.E., Chow L.Q., Vokes E.E., Felip E., Holgado E. (2015). Nivolumab versus Docetaxel in Advanced Nonsquamous Non-Small-Cell Lung Cancer. N. Engl. J. Med..

[6] Brahmer J., Reckamp K.L., Baas P., Crinò L., Eberhardt W.E., Poddubskaya E., Antonia S., Pluzanski A., Vokes E.E., Holgado E. (2015). Nivolumab versus Docetaxel in Advanced Squamous-Cell Non-Small-Cell Lung Cancer. N. Engl. J. Med..

[7] Herbst R.S., Baas P., Kim D.W., Felip E., Perez-Gracia J.L., Han J.Y., Molina J., Kim J.H., Arvis C.D., Ahn M.J. (2016). Pembrolizumab versus docetaxel for previously treated, PD-L1-positive, advanced non-small-cell lung cancer (KEYNOTE-010): A randomised controlled trial. Lancet.

[8] Reck M., Rodriguez-Abreu D., Robinson A.G., Hui R., Csoszi T., Fulop A., Gottfried M., Peled N., Tafreshi A., Cuffe S. (2016). Pembrolizumab versus Chemotherapy for PD-L1-Positive Non-Small-Cell Lung Cancer. N. Engl. J. Med..

[9] Rittmeyer A., Barlesi F., Waterkamp D., Park K., Ciardiello F., von Pawel J., Gadgeel S.M., Hida T., Kowalski D.M., Dols M.C. (2017). Atezolizumab versus docetaxel in patients with previously treated non-small-cell lung cancer (OAK): A phase 3, open-label, multicentre randomised controlled trial. Lancet.

[10] Bellmunt J., de Wit R., Vaughn D.J., Fradet Y., Lee J.L., Fong L., Vogelzang N.J., Climent M.A., Petrylak D.P., Choueiri T.K. (2017). Pembrolizumab as Second-Line Therapy for Advanced Urothelial Carcinoma. N. Engl. J. Med..

[11] Motzer R.J., Escudier B., McDermott D.F., George S., Hammers H.J., Srinivas S., Tykodi S.S., Sosman J.A., Procopio G., Plimack E.R. (2015). Nivolumab versus Everolimus in Advanced Renal-Cell Carcinoma. N. Engl. J. Med..

[12] Balar A.V., Castellano D., O’Donnell P.H., Grivas P., Vuky J., Powles T., Plimack E.R., Hahn N.M., de Wit R., Pang L. (2017). First-line pembrolizumab in cisplatin-ineligible patients with locally advanced and unresectable or metastatic urothelial cancer (KEYNOTE-052): A multicentre, single-arm, phase 2 study. Lancet Oncol..

[13] Michot J.M., Bigenwald C., Champiat S., Collins M., Farbonnel F., Postel-Vinay S., Berdelou A., Varga A., Bahleda R., Hollebecque A. (2016). Immune-related adverse events with immune checkpoint blockage: A comprehensive review. Eur. J. Cancer.

[14] Pauken K.E., Dougan M., Rose N.R., Lichtman A.H., Sharpe A.H. (2019). Adverse Events Following Cancer Immunotherapy: Obstacles and Opportunities. Trends Immunol..

[15] Wang Y., Zhou S., Yang F., Qi X., Wang X., Guan X., Shen C., Duma N., Vera Aguilera J., Chintakuntlawar A. (2019). Treatment-Related Adverse Events of PD-1 and PD-L1 Inhibitors in Clinical Trials: A Systematic Review and Meta-analysis. JAMA Oncol..

[16] Haanen J.B.A.G., Carbonnel F., Robert C., Kerr K.M., Peters S., Larkin J., Jordan K., ESMO Guidelines Committee (2017). Management of toxicities from immunotherapy: ESMO Clinical Practice Guidelines for diagnosis, treatment and follow-up. Ann. Oncol..

[17] Spain L., Diem S., Larkin J. (2016). Management of toxicities of immune checkpoint inhibitors. Cancer Treat. Rev..

[18] Khan Z., Hammer C., Carroll J., Di Nucci F., Acosta S.L., Maiya V., Bhangale T., Hunkapiller J., Mellman I., Albert M.L. (2021). Genetic variation associated with thyroid autoimmunity shapes the systemic immune response to PD-1 checkpoint blockade. Nat. Commun..

[19] Haanen J., Ernstoff M.S., Wang Y., Menzies A.M., Puzanov I., Grivas P., Larkin J., Peters S., Thompson J.A., Obeid M. (2020). Autoimmune diseases and immune-checkpoint inhibitors for cancer therapy: Review of the literature and personalized risk-based prevention strategy. Ann. Oncol..

[20] Harjutsalo V., Sund R., Knip M., Groop P.H. (2013). Incidence of type 1 diabetes in Finland. JAMA.

[21] Virta L.J., Saarinen M.M., Kolho K.L. (2017). Declining trend in the incidence of biopsy-verified coeliac disease in the adult population of Finland, 2005-2014. Aliment. Pharmacol. Ther..

[22] Mäkimattila S., Harjutsalo V., Forsblom C., Groop P.H., FinnDiane Study Group (2020). Every Fifth Individual With Type 1 Diabetes Suffers From an Additional Autoimmune Disease: A Finnish Nationwide Study. Diabetes Care.

[23] Freeman-Keller M., Kim Y., Cronin H., Richards A., Gibney G., Weber J.S. (2016). Nivolumab in Resected and Unresectable Metastatic Melanoma: Characteristics of Immune-Related Adverse Events and Association with Outcomes. Clin. Cancer Res..

[24] Sanlorenzo M., Vujic I., Daud A., Algazi A., Gubens M., Luna S.A., Lin K., Quaglino P., Rappersberger K., Ortiz-Urda S. (2015). Pembrolizumab Cutaneous Adverse Events and Their Association with Disease Progression. JAMA Dermatol.

[25] Berner F., Bomze D., Diem S., Ali O.H., Fässler M., Ring S., Niederer R., Ackermann C.J., Baumgaertner P., Pikor N. (2019). Association of Checkpoint Inhibitor-Induced Toxic Effects With Shared Cancer and Tissue Antigens in Non-Small Cell Lung Cancer. JAMA Oncol..

[26] Iivanainen S., Alanko T., Vihinen P., Konkola T., Ekstrom J., Virtanen H., Koivunen J. (2020). Follow-Up of Cancer Patients Receiving Anti-PD-(L)1 Therapy Using an Electronic Patient-Reported Outcomes Tool (KISS): Prospective Feasibility Cohort Study. JMIR Form. Res..

[27] Iivanainen S., Ekström J., Virtanen H., Kataja V.V., Koivunen J.P. (2022). Predicting Objective Response Rate (ORR) in Immune Checkpoint Inhibitor (ICI) Therapies with Machine Learning (ML) by Combining Clinical and Patient-Reported Data. Appl. Sci..

[28] Nohynek H., Jokinen J., Partinen M., Vaarala O., Kirjavainen T., Sundman J., Himanen S.L., Hublin C., Julkunen I., Olsen P. (2012). AS03 Adjuvanted AH1N1 Vaccine Associated with an Abrupt Increase in the Incidence of Childhood Narcolepsy in Finland. PLoS ONE.

[29] Bergman P., Adori C., Vas S., Kai-Larsen Y., Sarkanen T., Cederlund A., Agerberth B., Julkunen I., Horvath B., Kostyalik D. (2014). Narcolepsy patients have antibodies that stain distinct cell populations in rat brain and influence sleep patterns. Proc. Natl. Acad. Sci. USA.

[30] https://www.ema.europa.eu/en/documents/rmp-summary/nivolumab-bms-epar-risk-management-plan-summary_en.pdf.

[31] https://www.fimea.fi/documents/542809/835259/29889_Keytruda_RMP_summary-EN.pdf.

[32] Pasello G., Pavan A., Attili I., Bortolami A., Bonanno L., Menis J., Conte P., Guarneri V. (2020). Real world data in the era of Immune Checkpoint Inhibitors (ICIs): Increasing evidence and future applications in lung cancer. Cancer Treat. Rev..

[33] Raschi E., Gatti M., Gelsomino F., Ardizzoni A., Poluzzi E., De Ponti F. (2020). Lessons to be Learnt from Real-World Studies on Immune-Related Adverse Events with Checkpoint Inhibitors: A Clinical Perspective from Pharmacovigilance. Targ. Oncol..

[34] Larkin J., Chiarion-Sileni V., Gonzalez R., Grob J.J., Rutkowski P., Lao C.D., Cowey C.L., Schadendorf D., Wagstaff J., Dummer R. (2019). Five-Year Survival with Combined Nivolumab and Ipilimumab in Advanced Melanoma. N. Engl. J. Med..

[35] Hellmann M.D., Paz-Ares L., Bernabe Caro R., Zurawski B., Kim S.W., Carcereny Costa E., Park K., Alexandru A., Lupinacci L., de la Mora Jimenez E. (2019). Nivolumab plus Ipilimumab in Advanced Non-Small-Cell Lung Cancer. N. Engl. J. Med..

[36] Doherty P.C., Zinkernagel R.M. (1975). A biological role for the major histocompatibility antigens. Lancet.

[37] Brooks A.G., Boyington J.C., Sun P.D. (2000). Natural killer cell recognition of HLA class I molecules. Rev. Immunogenet..

[38] Aptsiauri N., Cabrera T., Garcia-Lora A., Lopez-Nevot M.A., Ruiz-Cabello F., Garrido F. (2007). MHC class I antigens and immune surveillance in transformed cells. Int. Rev. Cytol..

[39] Lozano A.X., Chaudhuri A.A., Nene A., Bacchiocchi A., Earland N.E., Vesely M.D., Usmani A., Turner B.E., Steen C.B., Luca B.A. (2022). T cell characteristics associated with toxicity to immune checkpoint blockade in patients with melanoma. Nat. Med..

[40] Ikeda T., Ishihara H., Nemoto Y., Tachibana H., Fukuda H., Yoshida K., Takagi T., Iizuka J., Hashimoto Y., Ishida H. (2021). Prognostic impact of immune-related adverse events in metastatic renal cell carcinoma treated with nivolumab plus ipilimumab. Urol Oncol..

[41] Johnson D.B., McDonnell W.J., Gonzalez-Ericsson P.I., Al-Rohil R.N., Mobley B.C., Joe-Elie S., Wang D.Y., Sanchez V., Wang Y., Chastain C.A. (2019). A case report of clonal EBV-like memory CD4+ T cell activation in fatal checkpoint inhibitor-induced encephalitis. Nat. Med..

[42] Dubin K., Callahan M.K., Ren B., Khanin R., Viale A., Ling L., No D., Gobourne A., Littmann E., Huttenhower C. (2016). Intestinal microbiome analyses identify melanoma patients at risk for checkpointblockade-induced colitis. Nat. Commun..

[43] Iwama S., De Remigis A., Callahan M.K., Slovin S.F., Wolchok J.D., Caturegli P. (2014). Pituitary expression of CTLA-4 mediates hypophysitis secondary to administration of CTLA-4 blocking antibody. Sci. Transl. Med..

[44] Johnson D.B., Nebhan C.A., Moslehi J.J., Balko J.M. (2022). Immune-checkpoint inhibitors: Long-term implications of toxicity. Nat. Rev. Clin. Oncol..

[45] Zheng Y., Kim R., Yu T., Gayle J.A., Wassel C.L., Dreyfus J., Phatak H., George S. (2021). Real-World Clinical and Economic Outcomes in Selected Immune-Related Adverse Events Among Patients with Cancer Receiving Immune Checkpoint Inhibitors. Oncologist..

[46] Wang D.Y., Salem J.E., Cohen J.V., Chandra S., Menzer C., Ye F., Zhao S., Das S., Beckermann K.E., Ha L. (2018). Fatal Toxic Effects Associated With Immune Checkpoint Inhibitors: A Systematic Review and Meta-analysis. JAMA Oncol..

